# Distal Junctional Failures in Degenerative Thoracolumbar Hyperkyphosis

**DOI:** 10.1111/os.13973

**Published:** 2024-02-21

**Authors:** Yongqiang Wang, Junyu Li, Yu Xi, Yan Zeng, Miao Yu, Zhuoran Sun, Yinghong Ma, Zhongjun Liu, Zhongqiang Chen, Weishi Li

**Affiliations:** ^1^ Department of Orthopedics Peking University Third Hospital Beijing China; ^2^ Beijing Key Laboratory of Spinal Disease Research Peking University Third Hospital Beijing China; ^3^ Engineering Research Center of Bone and Joint Precision Medicine, Ministry of Education Peking University Third Hospital Beijing China; ^4^ Peking University Health Science Center Beijing China

**Keywords:** Degenerative thoracolumbar hyperkyphosis, Distal junctional failures, Fusion range, Risk factor, Sagittal stable vertebra

## Abstract

**Objective:**

Degenerative thoracolumbar hyperkyphosis (DTH) is a disease that negatively affects individual health and requires surgical intervention, yet the ideal surgical approach and complications, especially distal junctional failures (DJF), remain poorly understood. This study aims to investigate DJF in DTH and to identify the risk factors for DJF so that we can improve surgical decision‐making, and advance our knowledge in the field of spinal surgery to enhance patient outcomes.

**Methods:**

This study retrospectively reviewed 78 cases (late osteoporotic vertebral compression fracture [OVCF], 51; Scheuermann's kyphosis [SK], 17; and degenerative disc diseases [DDD], 10) who underwent corrective surgery in our institute from 2008 to 2019. Clinical outcomes were assessed using health‐related quality of life (HRQOL) measures, including the visual analogue scale (VAS) scores for back and leg pain, the Oswestry disability index (ODI), and the Japanese Orthopaedic Association (JOA) scoring system. Multiple radiographic parameters, such as global kyphosis (GK) and thoracolumbar kyphosis (TLK), were assessed to determine radiographic outcomes. Multivariate logistic regression analysis was employed to identify the risk factors associated with DJF.

**Results:**

HRQOL improved, and GK, TLK decreased at the final follow‐up, with a correction rate of 67.7% and 68.5%, respectively. DJF was found in 13 of 78 cases (16.7%), two cases had wedging in the disc (L3‐4) below the instrumentation, one case had a fracture of the lowest instrumented vertebrae (LIV), one case had osteoporotic fracture below the fixation, nine cases had pull‐out or loosening of the screws at the LIV and three cases (23.1%) required revision surgery. The DJF group had older age, lower computed tomography Hounsfield unit (CT HU), longer follow‐up, more blood loss, greater preoperative sagittal vertical axis (SVA), and poorer postoperative JOA and VAS scores (back). The change in TLK level was larger in the non‐DJF group. Post‐sagittal stable vertebrae (SSV) moved cranially compared with pre‐SSV.

**Conclusion:**

Age, CT HU, length of follow‐up, estimated blood loss, and preoperative SVA were independent risk factors for DJF. We recommend fixation of the two vertebrae below the apex vertebrae for DTH to minimize surgical trauma.

## Graphical abstract

We retrospectively reviewed patients with degenerative thoracolumbar hyperkyphosis (DTH) underwent corrective surgery in our institute, and found that age, computed tomography Hounsfield unit (CT HU), length of follow‐up, estimated blood loss, and preoperative SVA were independent risk factors for development of distal junctional failures (DJF). SSV, SSV−, and SSV+ groups showed no statistically significant differences in terms of the incidence of DJF, which indicated that fusion to SSV or more caudally could not inhibit the occurrence of DJF. We recommend fixation of the two vertebrae below the apex vertebrae for DTH to minimize surgical trauma.

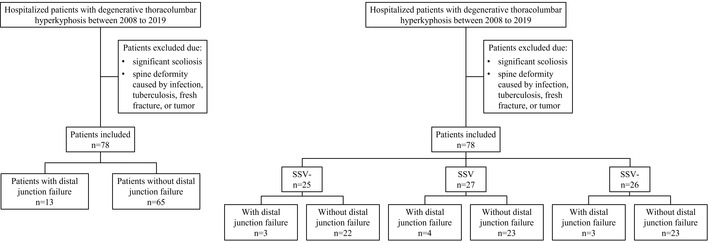

## Introduction

Thoracic kyphosis is the normal curvature of the thoracic spine in the sagittal plane, which tends to increase with age. In younger adults, the range of curvature is between 20° and 30°, using the Cobb angle as a measurement of kyphosis. In adults over the age of 40, the curvature may gradually increase above 40°.^1^ Hyperkyphosis refers to excessive curvature of the thoracic spine. However, there is currently no universally accepted definition for hyperkyphosis. The cause of hyperkyphosis is multifactorial and involves an interaction between degenerative changes, vertebral compression fractures, muscular weakness, and altered biomechanics.^2^ It has been reported with an estimated prevalence ranging from 20% to 40%,^2^, ^3^ and hyperkyphosis may negatively impact individual's health.

Progressive kyphosis, refractory pain, and neurological compromise were the most relevant findings indicative of surgical treatment.^3^ Surgical intervention for thoracic kyphosis in an aging population is challenging because patients often have combined comorbidities and poor bone quality, and the ideal surgical strategy remains controversial, especially on the selection of fusion levels. Usually, long segment fusions are required. Ailon *et al*. advocated that sacropelvic fixation should be considered for any fusion that extends proximally to T12 or above.^4^ Sagittal stable vertebra (SSV) refers to the most proximal vertebra touched by the posterior sacral vertical line, and it is commonly used as a reference for fusion level in corrective spinal surgery. Wang *et al*. reported that fusion to SSV could limit the development of distal junctional mechanical complications in thoracolumbar kyphosis secondary to late osteoporotic vertebral compression fracture (OVCF).^5^ While Cecchinato *et al*. utilized short segment fusion with anterior corpectomy to treat post‐traumatic thoracolumbar deformity.^6^

Corrective spinal surgery is commonly associated with complications, which could lead to revision surgery. Junctional mechanical complications after spinal fusion surgery have been widely reported during the past decades. Junctional mechanical complication is a broad term including a range of conditions, such as proximal junctional kyphosis (PJK), proximal junctional failure (PJF), distal junctional kyphosis (DJK), distal junctional failure (DJF), and junctional scoliosis (JS).^7^

In contrast to proximal junctional failure, distal junctional failure has received less attention in the literature.^8^ Arlet and Aebi have described the most common DJF modes (Table 1).^9^ DJF often gives rise to a spectrum of distressing symptoms, encompassing persistent pain, compromising neurological function, and a gradual advancement of deformity. These manifestations contribute to a substantial deterioration in the patient's quality of life. Moreover, in more severe instances, the escalating impact of DJF necessitates a decisive intervention in the form of revision surgery, aimed at rectifying the exacerbated condition. This situation underscores the imperative for monitoring and proactive management strategies to mitigate the distress and potential complications associated with DJF. Previous publications have reported that selection of lowest instrumented vertebra (LIV) was a risk factor for DJF.^10^ Since Cho *et al*.^11^ reported that LIV stopped at the SSV could prevent DJK, many authors have reported similar results.^5^, ^12^, ^13^ While during the clinical practice, we have noticed that DJF might occur even when LIV stopped at the SSV.

**TABLE 1:** Modes of DJF.^9^

1. Progressive loss of lumbar lordosis, disc degeneration with loss of height
2. Acute wedging in disc below instrumentation
3. Fracture of distal instrumented vertebra
4. Osteoporotic fracture below long rigid fixation
5. Failure of instrumentation at most distal level
6. Spinal stenosis and/or segmental instability below instrumentation

Abbreviation: DJF, distal junctional failures.

Thus, we retrospectively reviewed patients with thoracic kyphosis who underwent corrective surgery in our institute, excluding those caused by infection, tuberculosis and fresh fracture. We found that apex vertebrae of all cases located at T10‐L2 area, so we named the cohort degenerative thoracolumbar hyperkyphosis (DTH). This study aimed to: (i) assess the effectiveness of surgical intervention by evaluating clinical and radiographic parameters; (ii) investigate the incidence of DJF and identify the risk factors for DJF in DTH patients; and (iii) investigate the impact of surgical procedures, especially the selection of LIV, on prognosis of DTH patients. Most published literature on DJF focuses on adolescent idiopathic scoliosis (AIS) and adolescent Scheuermann's kyphosis (SK). To the best of our knowledge, this is the first study to focus on DJF in a DTH cohort.

## Materials and Methods

### Inclusion Criteria and Exclusion Criteria

We retrospectively reviewed the electronic database of our hospital, and identified 78 patients with thoracolumbar hyperkyphosis among cases from 2008 to 2019. The etiology included 51 cases of late OVCF, 17 cases of SK, and 10 cases of degenerative disc diseases (DDD).

The inclusion criteria were as follows: (i) age >45 years; (ii) presence of thoracolumbar hyperkyphosis of >30°; (iii) minimum 2‐year follow‐up; and (iv) availability of complete radiographs. Informed consent was obtained from all patients.

The exclusion criteria were as follows: (i) presence of scoliosis with a Cobb angle > of 10°; (ii) spine deformity caused by infection, tuberculosis, fresh fracture, or tumor; or (iii) history of spinal surgery.

### Diagnosis Criteria

The diagnosis of thoracolumbar hyperkyphosis caused by OVCFs is mainly based on radiography.^14^ Diagnostic criteria are as follows: (i) presence of thoracolumbar hyperkyphosis of >30°; (ii) a decrease in vertebral body height of at least 20% or a 4‐mm reduction from baseline height; and (iii) classic radiographic characteristics including anterior wedging of vertebrae with vertebral collapse, vertebral endplate irregularity, and general demineralization.

The diagnostic criteria of thoracolumbar hyperkyphosis caused by SK include: (i) presence of thoracolumbar hyperkyphosis of >30°; (ii) wedging of at least three adjacent vertebral bodies by 5° or more; (iii) irregular endplates with Schmorl's nodes in multiple adjacent vertebral bodies; and (iv) no other pathological changes.^15^

The diagnosis of thoracolumbar hyperkyphosis caused by DDD is based on medical history, clinical manifestations and radiography.^16^ Diagnostic criteria are as follows: (i) presence of thoracolumbar hyperkyphosis of >30°; (ii) classic radiographic appearances including decrease in disc height, annular tears, signs of disc degeneration (such as decreased signal on T2‐weighted images), and endplate changes; and (iii) no other pathological changes.

The diagnosis of DJF is based on the common modes of DJF described by Arlet and Aebi (Table 1).^9^ Diagnostic criteria include: (i) progressive loss of lumbar lordosis, disc degeneration with loss of height; (ii) acute wedging in disc below instrumentation; (iii) fracture of distal instrumented vertebra; (iv) osteoporotic fracture below long rigid fixation; (v) failure of instrumentation at most distal level; and (vi) spinal stenosis and/or segmental instability below instrumentation.

### Surgical Strategy

All procedures were performed by the same surgical team. All the patients were operated through the single posterior approach, using complete pedicle screws and titanium rods fixation, with or without osteotomy. The fusion level tried to be symmetric above and below the apex, usually two or three vertebrae above and below the apex were involved in the fusion; however, when the planned upper‐most instrumented vertebra (UIV) had a fracture, proximal fixation should be extended. When DDD contributes to the thoracolumbar hyperkyphosis, necessitating neural decompression, the distal fixation level might be extended accordingly.^17^

Another critical aspect of the surgery is the choice of osteotomy. The osteotomy sites were usually chosen at the apex of the deformity, and posterior column osteotomy (PCO), pedicle subtraction osteotomy (PSO), modified grade 4 osteotomy, and vertebral column resection (VSR) were chosen depending on the severity and pattern of the curvature.^18^, ^19^ PCO includes Smith–Petersen osteotomy (SPO) and Ponte osteotomy. SPO involves resection of the lower facet joint and joint capsule. Ponte osteotomy involves the removal of the upper and lower facet joints, as well as the ligamentum flavum, accompanied by selective excision of the lamina and spinous process. Generally, the resection of approximately 1 mm in the sagittal plane corresponds to the correction of around 1° of deformity. This approach is particularly suited for gradual corrections, such as SK.

PSO is typically categorized as SRS‐Schwab grade 3, involves a wedge resection of a portion of the vertebra and its posterior elements while preserving the intervertebral discs and adjacent endplates. An extended PSO corresponds to grade 4 and involves the complete removal of these tissues. PSO is particularly suitable for patients with sagittal imbalance exceeding 10–12 cm, with a potential for correcting angles of up to 30°. In practice since 1996, we have utilized the modified grade 4 osteotomy to treat post‐traumatic thoracolumbar kyphosis.

The osteotomy site is meticulously closed to facilitate “bone‐to‐bone” fusion. In cases where adequate closure is not achieved, the implementation of cages and titanium mesh augmentation might be considered.

### Clinical and Radiographic Parameter Measurements

The visual analogue scale (VAS) scores for the back and leg, Oswestry disability index (ODI), and the Japanese Orthopaedic Association (JOA) scoring system were used to assess the patients' health‐related quality of life (HRQOL).

Spinopelvic parameters including pelvic incidence (PI), sacral slope (SS), lumbar lordosis (LL), and global kyphosis (GK), measured from the upper end vertebrae to the lower end vertebra of kyphosis according to the Cobb method; thoracic kyphosis (TK), measured from T5 to T12; thoracolumbar kyphosis (TLK), measured from T10 to L2; T1 pelvic angle (T1PA); the angle between the superior endplate of the lowest instrumented vertebrae (LIV) and the inferior endplate of the adjacent distal vertebrae, distal junctional angle (DJA); and the distance from the center of the LIV to the posterior sacral vertical line (LIV‐PSVL) were obtained from long‐cassette lateral radiographs before surgery, immediately after surgery, and at the final follow‐up.

We reviewed the preoperative three‐dimensional reconstructive dual‐source computed tomography (Siemens, SOMATOM DEFINITION, Forchheim, Germany, tube voltage 120 kV) images of L1 by using picture archiving and communication system (PACS). CT Hounsfield (HU) value was measured by placing an oval region of interest over an axial image of the vertebral mid‐body through L1. A CT HU value < of 110 was considered indicative for osteoporosis.^20^

All parameters were measured separately by two expert spine surgeons who were independent of the operations.

### Statistical Analysis

Clinical and radiographic data were analyzed using the Statistical Package for Social Sciences version 22.0 (SPSS, Inc., Chicago, IL, USA). Descriptive statistics are presented as means with standard deviations (SDs), and count data are presented as numbers. Statistical significance was set at *p* < 0.05. A paired *t*‐test was used to analyze postoperative changes in the parameters. The independent *t*‐test and Mann–Whitney *U*‐test were used to analyze the differences in continuous variables between the groups. Fisher's exact test and chi‐squared test were used to examine the differences among categorical variables. Variables with a *p*‐value of < 0.05 in intergroup comparison were included for further stepwise multivariate logistic regression using the likelihood ratio method to eliminate confounding factors. A significant factor whose 95% confidence interval of the odds ratio did not include unity was identified as an independent risk factor.

## Results

### General Information

Seventy‐eight patients (16 men, 62 women) with a mean age of 61.8 ± 6.4 (range, 47–77) years were included. The mean follow‐up period was 46.4 ± 18.1 (range, 26–88) months (Table 2). Among the study population, 9 patients underwent PCO, and 69 patients underwent PSO or modified grade 4 osteotomy. The average fusion level was 5.2 ± 1.9. The operation time was 262 ± 71 (range, 142–544) min, and the estimated mean blood loss was 1005 ± 663 mL (range, 200–3700). The LIV ranged from L1 to L5. As for complications, among the included cohort, 42 patients had osteoporosis, 22 had hypertension, and five had diabetes. The average number of other complications (including orthopedic, cardiovascular, neurological and digestive diseases, and postoperative complications due to other causes) per patient was 1.0 ± 1.5.

**TABLE 2:** General information of the whole cohort.

Age (years)	61.8 ± 6.4
Gender (F/M)	62/16
Body mass index (Kg/m^2^)	25.2 ± 3.5
BMD *T*‐value	−2.6 ± 1.6
CT HU	112.1 ± 45.5
Osteoporosis (Y/N)	42/36
Hypertension (Y/N)	22/56
Diabetes (Y/N)	5/73
Other complications	1.1 ± 1.5
Fusion levels	5.2 ± 1.9
Follow‐up (months)	46.4 ± 18.1
Osteotomy	
PCO	9
Grade 3/4	69
LIV	
OV + 2	22
OV + 3	36
OV + more than 3 levels	20
Operation time (minutes)	261.7 ± 70.6
Estimated blood loss (mL)	1005.1 ± 663.1
GK (**°**)	
Preoperatively	60.0 ± 17.2
Postoperatively	19.9 ± 11.8
At final follow‐up	21.0 ± 12.1
*p*‐value	<0.001*
Correction rate of GK (%)	**67.7%**
TK (**°**)	
Preoperatively	37.4 ± 17.4
Postoperatively	28.3 ± 18.7
At final follow‐up	29.8 ± 12.4
*p*‐value	<0.001*
TLK (**°**)	
Preoperatively	43.8 ± 15.9
Postoperatively	12.3 ± 10.5
At final follow‐up	13.8 ± 9.6
Change in TLK	30.9 ± 18.1
*p*‐value	<0.001*
Correction rate of TLK (%)	**68.5%**
LL (**°**)	
Preoperatively	33.4 ± 21.9
Postoperatively	39.5 ± 17.9
At final follow‐up	36.6 ± 14.1
*p*‐value	0.313
PI (**°**)	
Preoperatively	46.9 ± 13.7
Postoperatively	48.5 ± 19.0
At final follow‐up	50.3 ± 14.1
*p*‐value	0.553
PT (**°**)	
Preoperatively	26.3 ± 13.5
Postoperatively	20.8 ± 11.6
At final follow‐up	25.4 ± 12.9
*p*‐value	0.490
SS (**°**)	
Preoperatively	22.1 ± 10.8
Postoperatively	31.4 ± 34.4
At final follow‐up	25.1 ± 10.8
*p*‐value	0.331
SVA (mm)	
Preoperatively	44.0 ± 45.7
Postoperatively	27.7 ± 27.7
At final follow‐up	28.6 ± 27.9
*p*‐value	<0.001*
Change in SVA	6.1 ± 38.0
T1PA (**°**)	
Preoperatively	30.1 ± 16.8
Postoperatively	19.2 ± 10.3
At final follow‐up	20.5 ± 12.5
*p*‐value	<0.001*
LIV‐PSVL (mm)	
Preoperatively	−5.6 ± 22.5
Postoperatively	13.9 ± 19.9
At final follow‐up	11.1 ± 19.1
*p*‐value	<0.001*
ODI	
Preoperatively	42.7 ± 17.4
At final follow‐up	9.6 ± 8.6
*p*‐value	<0.001*
JOA	
Preoperatively	17.7 ± 4.6
At final follow‐up	26.4 ± 2.3
*p*‐value	<0.001*
VAS (back)	
Preoperatively	6.1 ± 1.8
Postoperatively	0.7 ± 1.1
*p*‐value	<0.001*
VAS (leg)	
Preoperatively	1.4 ± 2.2
At final follow‐up	0.1 ± 0.4
*p*‐value	<0.001*

*Note*: Boldface values indicate *p* < 0.05.

Abbreviations: BMD, bone mineral density; GK, general kyphosis; Grade 3/4, pedicle subtraction osteotomy and modified grade 4 osteotomy; JOA, Japanese Orthopaedic Association; LIV, lowest instrumented vertebrae; LIV‐PSVL, the distance from the center of the LIV to the posterior sacral vertical line; LL, lumbar lordosis; ODI, oswestry disability index; OV, osteotomized vertebrae; PCO, posterior column osteotomy; PI, pelvic incidence; PT, pelvic tilt; SS, sacral slope; SVA, sagittal vertical axis; T1PA, T1 pelvic angle; TK, thoracic kyphosis; TLK, thoracolumbar kyphosis; VAS, visual analogue scale.

### Clinical and Radiographic Parameters

Back and leg pain were successfully relieved as evidenced by decrease in mean VAS scores of 6.1 ± 1.8 (range, 2–10) and 1.4 ± 2.2 (range, 0–8) preoperatively to 0.7 ± 1.1 (range, 0–6) and 0.1 ± 0.4 (range, 0–4) postoperatively, respectively. The neurological deficit improved as indicated by a JOA score that improved from 17.7 ± 4.6 (range, 7–26) preoperatively to 26.4 ± 2.3 (range, 11–29), whereas ODI decreased from 42.7 ± 17.4 (range, 12–90) preoperatively to 9.6 ± 8.6 (range, 0–64) postoperatively (Table 2). GK and TLK decreased from 60.0 ± 17.2 (range, 30–118) and 43.8 ± 15.9 (range, 8–55) to 19.9 ± 11.8 (range, 3.1–52.3) and 12.3 ± 10.5 (range, 2.1–54.9) after surgery but worsened to 21.0 ± 12.1 (range, 4.5–49.8) and 13.8 ± 9.6 (range, 3.7–43.2) at the final follow‐up, with a correction rate of 67.7% and 68.5%, respectively. TK, SVA, and T1PA in the entire cohort significantly decreased, and LIV‐PSVL, LL and SS slightly increased (Table 2).

### Comparison between Patients with or without DJF

**FIGURE 1:**
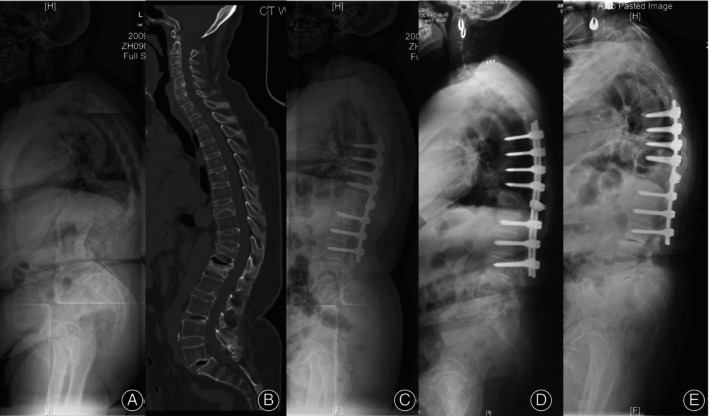
A 65‐year‐old woman presented with severe back pain. (A). The standing long‐cassette lateral radiographs showed thoracolumbar hyper‐kyphosis arising from osteoporotic vertebral compression fracture (OVCF) and L5 spondylolisthesis, global kyphosis (GK) was 82.3°and thoracolumbar kyphosis (TLK) was 53.4°, sagittal stable vertebrae (SSV) located at L3. (B) Computed tomography revealed T9, T11, T12, and L1 fractures. (C) Postoperative lateral x‐ray showed T7‐L3 fixation and T11‐12 modified grade 4 osteotomy, GK decreased to 32.5°and TLK decreased to 6.2°, SSV moved cranially to L2. (D) Wedging in L34 disc was detected at 3‐years' follow‐up. (E) Degenerative changes progressed in L34 disc at 7‐years' follow‐up, GK and TLK was 55.1° and 10.6°, respectively.

**FIGURE 2:**
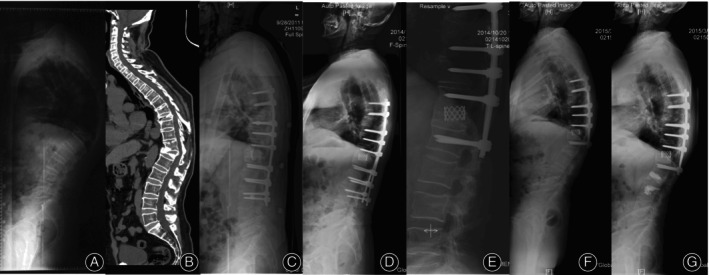
A 70‐year‐old man present with back pain, visual analogue scale (VAS) 8. (A) Lateral x‐ray showed thoracolumbar hyper‐kyphosis arising from T12 fracture, global kyphosis (GK) was 98°, thoracolumbar kyphosis (TLK) was 42.9°, sagittal stable vertebrae (SSV) located at L3. (B) Computed tomography revealed T8 and T12 fracture. (C) Postoperative lateral x‐ray showed T6‐L3 fixation, GK and TLK was 37.4° and 4.9°, respectively. (D) Pull‐out of L2 and L3 screws were detected at 3‐years' follow‐up. (E) After revision surgery, L2 and L3 screws were removed. (F) Five months later, the patient had a L2 fracture after falling. (G) Reoperated with vertebroplasty in L2 and L3, GK and TLK was 52.9° and 15.8°, respectively.

The incidence of DJF was 16.7% (13/78), 2 cases had wedging in the disc (L3‐4) below the instrumentation (Figure 1), 1 case had a fracture of the LIV, one case had osteoporotic fracture below the fixation, 9 cases had pull‐out or loosening of the screws at the LIV (Figure 2). 3 patients (23.1%) required revision surgery due to severe pain or progressive deformities, one of whom (T9‐L3 fixation, group SSV−) had loosening of L3 screw and underwent instrumentation extended to L5, one (T11‐L4 fixation, group SSV+) had pullout of L4 screws and was reoperated with S2‐alar iliac screws (S2AI), one (T6‐L3 fixation, group SSV) had pullout of L2 and L3 screws and was reoperated by removing L2 and L3 screws (Figure 2).

When comparing the clinical and radiographic outcomes between patients with and without DJF, the DJF group had older age, lower CT HU, longer follow‐up, more blood loss, greater preoperative SVA, and poorer postoperative ODI, JOA and VAS scores (back). The change in TLK level was larger in the non‐DJF group. Twenty‐two cases had an LIV two vertebrae below the apex, 36 cases had three vertebrae below the apex, and the remaining 20 cases had more than three vertebrae below the apex. The osteotomy pattern and number of vertebrae that were LIV away from the apex revealed no statistical differences between the two groups. In terms of complications, among the 13 patients in the DJF group, 9 had osteoporosis, 2 had hypertension, and 2 had diabetes, with an average of 1.1 ± 1.6 other complications. Among the 65 patients in the non‐DJF group, 33 had osteoporosis, 20 had hypertension, and three 3 had diabetes, with an average of 1.0 ± 1.2 other complications. Regarding complications, there was no significant difference between the DJF and non‐DJF groups. There were no significant differences between the DJF and non‐DJF groups concerning other characteristics such as gender, BMI, and BMD *T*‐value (Table 3).

**TABLE 3:** Comparison of radiographic and clinical assessment between patients with or without DJF

	Patients with DJF (*n* = 13)	Patients without DJF (*n* = 65)	*p*‐value
Age (years)	66.9 ± 5.3	60.8 ± 6.1	0.001*
Gender (F/M)	9/4	53/12	0.531
Body mass index (kg/m^2^)	26.5 ± 4.1	25.0 ± 3.4	0.168
BMD *T*‐value	−2.9 ± 1.6	−2.5 ± 1.6	0.460
CT HU	90.2 ± 57.0	116.6 ± 41.9	0.039*
Osteoporosis (Y/N)	9/4	33/32	0.223
Hypertension	2/11	20/45	0.330
Diabetes	2/11	3/62	0.192
Other complications	1.1 ± 1.6	1.0 ± 1.2	0.966
Fusion levels	5.2 ± 1.7	5.2 ± 1.9	0.914
Follow‐up (months)	56.3 ± 20.6	34.8 ± 12.5	0.010*
Osteotomy			
PCO	2	7	0.670
Grade 3/4	11	58	
LIV			
OV + 2	3	19	0.649
OV + 3	8	28	
OV+ more than 3 levels	2	18	
Operation time (minutes)	286.00 ± 68.18	256.83 ± 70.63	0.176
Estimated blood loss (mL)	1546.15 ± 1022.75	896.92 ± 511.35	0.043*
TK (°)			
Preoperatively	37.7 ± 17.1	37.4± 17.6	0.953
Postoperatively	30.5 ± 14.1	27.7± 19.7	0.641
At final follow‐up	29.4 ± 14.9	29.9± 11.9	0.907
*p*‐value	0.222	0.010*	
GK (°)			
Preoperatively	62.5 ± 20.9	59.5 ± 16.5	0.566
Postoperatively	17.3 ± 9.5	20.6 ± 12.2	0.365
At final follow‐up	20.9 ± 13.4	21.1 ± 12.0	0.968
*p*‐value	<0.001*	<0.001*	
Correction rate of GK (%)	73.2± 11.2	66.4 ± 18.3	0.204
TLK (°)			
Preoperatively	38.0 ± 17.1	45.1 ± 15.5	0.143
Postoperatively	14.7 ± 13.8	11.7 ± 9.7	0.473
At final follow‐up	14.9 ± 12.9	13.5 ± 8.8	0.652
Change in TLK	24.1 ± 16.3	31.9 ± 17.6	0.149
*p*‐value	<0.001*	<0.001*	
LL (°)			
Preoperatively	34.9 ± 19.0	33.1 ± 22.6	0.806
Postoperatively	36.7 ± 14.6	40.2 ± 18.7	0.531
At final follow‐up	35.9 ± 15.61	37.3 ± 14.0	0.472
*p*‐value	0.891	0.244	
PI (°)			
Preoperatively	51.1 ± 5.9	50.2 ± 12.1	0.711
Postoperatively	51.1 ± 6.0	52.0 ± 12.5	0.810
At final follow‐up	50.9 ± 6.1	49.5 ± 12.7	0.609
PT (°)			
Preoperatively	30.2 ± 5.8	29.1 ± 11.0	0.618
Postoperatively	20.4 ± 4.7	21.8 ± 8.9	0.635
At final follow‐up	20.0 ± 3.1	22.1 ± 9.8	0.255
SS (°)			
Preoperatively	20.9 ± 3.6	21.1 ± 7.7	0.865
Postoperatively	30.7 ± 4.4	30.3 ± 12.2	0.918
At final follow‐up	30.9 ± 5.5	27.5 ± 10.2	0.345
SVA (mm)			
Preoperatively	77.5 ± 55.0	36.3 ± 40.0	0.004*
Postoperatively	36.0 ± 20.5	25.6 ± 28.7	0.243
At final follow‐up	38.6 ± 23.0	28.5 ± 29.0	0.263
*p*‐value	0.040*	0.031*	
Change in SVA	38.9 ± 50.8	7.8 ± 36.3	0.016*
T1PA (°)			
Preoperatively	30.3 ± 18.7	30.0 ± 16.6	0.964
Postoperatively	24.0 ± 9.6	18.3 ± 10.1	0.085
At final follow‐up	24.3 ± 11.3	19.8 ± 12.5	0.279
*p*‐value	0.370	<0.001*	
LIV‐PSVL (mm)			
Preoperatively	−6.9 ± 33.1	−5.3 ± 20.2	0.877
Postoperatively	11.0 ± 28.9	14.5 ± 17.8	0.591
At final follow‐up	10.6 ± 23.9	11.1 ± 18.4	0.937
*p*‐value	0.196	<0.001*	
ODI			
Preoperatively	44.3 ± 15.3	42.4 ± 17.9	0.725
At final follow‐up	15.4 ± 10.1	8.5 ± 7.9	0.008*
*p*‐value	<0.001*	<0.001*	
JOA			
Preoperatively	16.5 ± 2.4	17.9 ± 4.9	0.137
At final follow‐up	24.5 ± 2.7	26.7 ± 2.1	0.016*
*p*‐value	<0.001*	<0.001*	
VAS (back)			
Preoperatively	7.2 ± 1.7	5.9 ± 1.8	0.020*
At final follow‐up	1.5 ± 1.5	0.5 ± 1.0	0.043*
*p*‐value	<0.001*	<0.001*	
VAS (leg)			
Preoperatively	1.2 ± 1.9	1.4 ± 2.4	0.674
Postoperatively	0.2 ± 0.6	0.1 ± 0.5	0.671
*p*‐value	0.020*	<0.001*	

*Note*: Boldface values indicate *p* < 0.05.

Abbreviations: BMD, bone mineral density; DJF, distal junction failure; GK, general kyphosis; Grade 3/4, pedicle subtraction osteotomy and modified grade 4 osteotomy; JOA, Japanese Orthopaedic Association; LIV, lowest instrumented vertebrae; LIV‐PSVL, the distance from the center of the LIV to the posterior sacral vertical line; LL, lumbar lordosis; ODI, Oswestry disability index; OV, osteotomized vertebrae; PCO, posterior column osteotomy; PI, pelvic incidence; PT, pelvic tilt; SS, sacral slope; SVA, sagittal vertical axis; T1PA, T1 pelvic angle; TK, thoracic kyphosis; TLK, thoracolumbar kyphosis; VAS, visual analogue scale.

Multivariate logistic regression analysis of possible risk factors for DJF revealed that age (odds ratio, 1.128; *p* = 0.017), CT HU (odds ratio, 0.986; *p* = 0.045), follow‐up (odds ratio, 1.072; *p* = 0.040), estimated blood loss (odds ratio, 1.001; *p* = 0.007), and preoperative SVA (odds ratio, 1.012; *p* = 0.036) were independent risk factors for DJF.

### Comparison between Groups SSV−, SSV and SSV+

**FIGURE 3:**
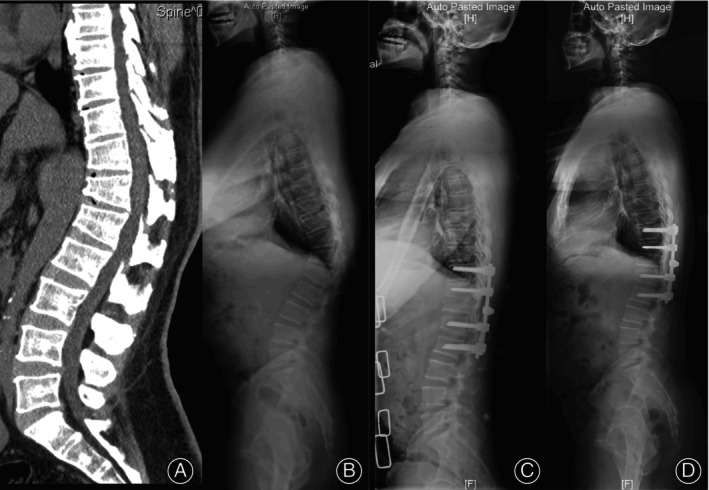
A 53‐year‐old man with degenerative thoracolumbar hyperkyphosis (DTH) arising from Scheuermann's kyphosis (SK). (A) Computed tomography showed wedging T11‐L2 vertebrae. (B) Lateral x‐ray showed global kyphosis (GK) and thoracolumbar kyphosis (TLK) were both 58.2°, sagittal stable vertebrae (SSV) located at L3. (C) Postoperative x‐ray showed fixation of two levels above and below the apex and modified grade 4 osteotomy, SSV moved cranially to L2, GK and TLK decreased to 21.9°. (D) Solid bony fusion could be seen without junctional complications at 2‐year follow‐up, GK and TLK maintained.

25 cases were assigned to group SSV− as the LIV was located cranially to the SSV. Similarly, 27 and 26 cases were respectively assigned to groups SSV and SSV+. Although BMD *T*‐value, fusion level, pattern of osteotomy, selection of LIV, and other demographic or radiographic differences were observed among these groups, the incidence of DJF showed no statistically significant differences (Table 4). Post‐SSV moved cranially compared with pre‐SSV, with a mean level of 0.8 ± 1.2 (Figure 3), also LIV‐PSVL increased from −5.6 to 13.9 mm (Table 2).

**TABLE 4:** Comparison of radiographic and clinical assessment between groups SSV and SSV− and between groups SSV and SSV+ (Ps: 0‐SSV−; 1‐SSV; 2‐SSV+).

	Group	Group	Group	*p*‐value (0,1)	*p*‐value (0,2)	*p*‐value (1,2)
	SSV−	SSV	SSV+			
	(*n* = 25)	(*n* = 27)	(*n* = 26)			
Age (years)	60.8 ± 6.4	61.3 ± 7.0	63.3 ± 5.5	0.943	0.340	0.510
Gender (M/F)	6/19	8/19	2/24	0.197	0.140	0.091
Body mass index (kg/m^2^)	25.3 ± 3.2	25.1 ± 3.6	25.3 ± 3.8	0.985	1.000	0.988
BMD *T*‐value	−1.7 ± 2.1	−2.8 ± 1.3	−2.9 ± 1.3	0.045*	0.021*	0.709
CT HU	122.6 ± 43.7	117.8 ± 47.4	97.0 ± 42.8	0.925	0.121	0.216
Osteoporosis (Y/N)	11/14	15/12	16/10	0.444		
Hypertension (Y/N)	8/17	7/20	7/19	0.875		
Diabetes (Y/N)	0/25	2/25	3/23	0.316		
Other complications	1.0 ± 1.5	1.0 ± 1.6	1.0 ± 1.2	0.984	0.947	0.989
Fusion levels	3.8 ± 1.3	5.6 ± 1.9	6.4 ± 1.7	<0.001*	<0.001*	0.053
Follow‐up (months)	41.1 ± 16.4	48.0 ± 20.9	47.8 ± 20.0	0.521	0.451	0.985
Osteotomy						
PCO	3	1	5	0.341	0.465	0.050
Grade 3/4	22	26	21			
DJF (Y/N)	3/22	4/23	6/20	1.000	0.465	0.676
LIV						
OV + 2	16	4	2	<0.001*		
OV + 3	7	18	11			
OV + more than 3 levels	2	5	13			
Operation time (minutes)	220.1 ± 51.1	264.9 ± 69.7	304.2 ± 80.5	0.022*	<0.001*	0.034*
Estimated blood loss (mL)	806.0 ± 506.3	812.9 ± 265.2	1388.7 ± 896.5	0.969	0.001*	0.001*
GK (°)						
Preoperatively	62.6 ± 15.1	61.2 ± 16.3	56.2 ± 22.4	0.781	0.201	0.330
Postoperatively	23.5 ± 11.5	21.2 ± 13.4	15.7 ± 9.1	0.777	0.065	0.777
At final follow‐up	26.6 ± 13.5	18.4 ± 11.7	18.4 ± 9.7	0.031*	0.032*	0.991
*p*‐value	<0.001*	<0.001*	<0.001*			
Correction rate (%)	63.5% ± 17.5%	67.6% ± 17.9%	71.7% ± 16.3%	0.440	0.122	0.422
TK (°)						
Preoperatively	43.0 ± 15.7	41.2 ± 14.7	28.2 ± 18.3	0.700	0.002*	0.007*
Postoperatively	31.1 ± 29.9	30.6 ± 11.3	23.2 ± 11.3	0.929	0.173	0.173
At final follow‐up	31.9 ± 13.4	28.6 ± 9.9	28.7 ± 13.8	0.397	0.417	0.980
*p*‐value	0.013*	0.001*	0.459			
TLK (°)						
Preoperatively	46.7 ± 13.5	44.9 ± 15.5	39.9 ± 18.2	0.679	0.134	0.281
Postoperatively	15.1 ± 9.1	12.7 ± 12.6	9.4 ± 8.6	0.457	0.078	0.260
At final follow‐up	17.4 ± 12.1	12.0 ± 8.0	11.8 ± 7.4	0.068	0.062	0.950
Change in TLK	29.2 ± 16.6	32.5 ± 16.2	29.6 ± 19.9	0.528	0.943	0.567
*p*‐value	<0.001*	<0.001*	<0.001*			
LL (°)						
Preoperatively	37.2 ± 16.8	33.0 ± 26.3	30.4 ± 22.0	0.517	0.296	0.691
Postoperatively	41.6 ± 14.0	37.5 ± 23.0	37.5 ± 23.0	0.457	0.755	0.657
At final follow‐up	37.6 ± 12.7	35.9 ± 12.7	36.4 ± 17.5	0.700	0.788	0.914
*p*‐value	0.004*	0.094	0.417			
PI (°)						
Preoperatively	45.1 ± 12.6	52.7 ± 11.6	53.0 ± 7.9	0.020*	0.016*	0.924
Postoperatively	45.5 ± 12.3	55.1 ± 12.1	53.8 ± 8.0	0.007*	0.024*	0.693
At final follow‐up	44.0 ± 12.5	52.9 ± 11.7	52.6 ± 9.4	0.025*	0.025*	0.947
PT (°)						
Preoperatively	23.7 ± 10.4	31.1 ± 9.8	32.7 ± 8.9	0.012*	0.002*	0.553*
Postoperatively	20.5 ± 9.3	22.3 ± 9.3	21.6 ± 6.0	0.482	0.688	0.768
At final follow‐up	19.9 ± 12.0	23.4 ± 7.6	22.3 ± 6.5	0.274	0.442	0.725
SS (°)						
Preoperatively	21.4 ± 7.8	21.6 ± 7.0	20.2 ± 6.9	0.918	0.585	0.512
Postoperatively	25.0 ± 13.5	32.8 ± 9.8	32.2 ± 9.6	0.027*	0.048*	0.864
At final follow‐up	24.1 ± 11.7	29.9 ± 7.3	30.4 ± 8.1	0.065	0.046*	0.900
SVA (mm)						
Preoperatively	21.2 ± 26.5	44.1 ± 32.9	46.9 ± 36.6	0.017*	0.016*	0.788
Postoperatively	17.3 ± 29.0	30.1 ± 26.3	31.7 ± 21.1	0.011*	0.049*	0.817
At final follow‐up	18.1 ± 28.5	30.8 ± 28.1	32.8 ± 20.4	0.035*	0.046*	0.728
*p*‐value	0.697	0.002*	0.006*			
Change in SVA	3.05 ± 31.80	13.80 ± 23.85	14.11 ± 36.12	0.035*	0.032*	0.973
T1PA (°)						
Preoperatively	26.0 ± 16.1	29.5 ± 16.4	34.7 ± 17.4	0.473	0.073	0.283
Postoperatively	21.0 ± 12.1	15.9 ± 10.6	20.5 ± 7.0	0.086	0.865	0.116
At final follow‐up	22.78 ± 17.3	17.5 ± 10.7	21.2 ± 12.4	0.154	0.661	0.312
*p*‐value	0.512	0.006*	0.001*			
LIV‐PSVL (mm)						
Preoperatively	−28.4 ± 18.1	1.4 ± 14.5	9.4 ± 12.6	<0.001*	<0.001*	0.048*
Postoperatively	−3.8 ± 20.1	21.9 ± 19.6	18.7 ± 17.8	<0.001*	<0.001*	0.328
At final follow‐up	−5.8 ± 21.0	21.3 ± 19.0	18.3 ± 15.9	<0.001*	<0.001*	0.425
*p*‐value	0.001*	<0.001*	0.001*			
ODI						
Preoperatively	38.2 ± 18.6	43.6 ± 16.5	44.3 ± 17.3	0.265	0.192	0.880
At final follow‐up	9.9 ± 8.1	8.3 ± 9.4	12.6 ± 12.4	0.571	0.338	0.117
*p*‐value	<0.001*	<0.001*	<0.001*			
JOA						
Preoperatively	18.6 ± 4.5	17.7 ± 4.6	17.1 ± 4.8	0.453	0.217	0.639
At final follow‐up	25.9 ± 2.1	27.0 ± 2.3	25.6 ± 3.6	0.154	0.716	0.062
*p*‐value	<0.001*	<0.001*	<0.001*			
VAS (back)						
Preoperatively	5.9 ± 1.5	6.0 ± 1.9	6.3 ± 2.1	0.877	0.459	0.549
Postoperatively	0.5 ± 0.9	0.6 ± 1.2	0.9 ± 1.3	0.815	0.176	0.252
*p*‐value	<0.001*	<0.001*	<0.001*			
VAS (leg)						
Preoperatively	1.5 ± 2.7	1.4 ± 1.9	1.5 ± 2.4	0.820	0.986	0.795
At final follow‐up	0.1 ± 0.6	0.1 ± 0.4	0.3 ± 0.9	0.962	0.346	0.311
*p*‐value	0.011*	0.013*	0.001*			

*Note*: Boldface values indicate *p* < 0.05.

Abbreviations: BMD, bone mineral density; DJF, distal junction failure; GK, general kyphosis; Grade 3/4, pedicle subtraction osteotomy and modified grade 4 osteotomy; JOA, Japanese Orthopaedic Association; LIV, lowest instrumented vertebrae; LIV‐PSVL, the distance from the center of the LIV to the posterior sacral vertical line; LL, lumbar lordosis; ODI, Oswestry disability index; OV, osteotomized vertebrae; PCO, posterior column osteotomy; PI, pelvic incidence; PT, pelvic tilt; SS, sacral slope; SSV, sagittal stable vertebra; SVA, sagittal vertical axis; T1PA, T1 pelvic angle; TK, thoracic kyphosis; TLK, thoracolumbar kyphosis; VAS, visual analogue scale.

### Perioperative Complications

One patient who underwent T9‐L3 fixation (SSV− group), developed wound dehiscence and cerebrospinal fluid leakage, necessitating a subsequent debridement surgery. The patient responded well to the treatment, and no further complications were reported. Another patient, who underwent T11‐L2 fixation (SSV− group), encountered cerebrospinal fluid leakage postoperatively. However, this complication was effectively managed through conservative therapy. Neither of the patients who experienced perioperative complications went on to develop DJF. The presence of perioperative complications does not appear to correlate with the subsequent DJF.

## Discussion

### Evaluation of Clinical and Radiographic Parameters

In this study, we retrospectively studied 78 thoracolumbar hyperkyphosis cases caused by different etiologies (OVCF, SK and DDD). The correction rates of GK and TLK were 67.7% and 68.5%, respectively. HRQOL got improvement, which showed satisfactory clinical outcomes (Table 2). SVA showed significant improvement, and T1PA decreased significantly, which was introduced by Protopsaltis^21^ as a novel measure of sagittal alignment that simultaneously accounts for both spinal inclination and pelvic retroversion. Theoretically, LL would decrease as a compensatory mechanism for the correction of TLK,^22^ while the current study showed that LL increased slightly at the final follow‐up. We speculate that the causes of this finding were as follows: (i) many OVCF cases have L1 fractures, and the collapsed upper endplate or even osteotomized L1 vertebrae may affect the measurement of LL; and (ii) long‐segment fusion in cases of DDD remodels lumbar lordosis.

### Incidence and Risk Factors of DJF

The incidence of DJF in this cohort was 16.7%; 3 cases (23.1%) required revision surgery, and most patients with DJF were non‐symptomatic and identified radiographically during regular follow‐up. DJF has received less attention in the literature, and most previous publications focused on AIS and SK. The incidence of DJK was reported as 0.2%–15%^19^, ^23^ and 12%–20.8%,^7^, ^11^, ^24^ respectively. To the best of our knowledge, this is the first study to focus on DJF in a DTH cohort, which excluded scoliosis cases. Cho *et al*.^25^ reported that preoperative sagittal decompensation was mostly due to complications at the distal segments, which are believed to correlate with HRQOL. Thus, patients with DJF had poorer clinical and radiographic outcomes with greater preoperative SVA and poorer postoperative ODI, JOA and VAS scores (back), and the change in TLK was larger in the non‐DJF group (Table 3).

Multivariate logistic regression analysis of possible risk factors for DJF revealed that age, CT HU, follow‐up (Tables 3 and 4), estimated blood loss, and preoperative SVA were independent risk factors for the occurrence of DJF. Older age and lower CT HU may combine with osteoporosis and weakness of the paraspinal muscles, which would decrease the resistance against the flexion moment arm; more estimated blood loss may indicate longer fusion and more disruption of paraspinal muscles; longer follow‐up indicates longer daily activities including sitting and bending; and greater SVA would increase the flexion moment arm, all of which contribute to the progression of DJF.^8^ Kwon *et al*.^26^ also found that osteoporosis contributes to the development of DJF, equivalent to that observed in the current study. Ghasemi *et al*.^12^ found that younger patients in the SK cohort were more likely to develop DJK, which differs from our observations, perhaps because of the heterogeneous cohorts.

In addressing the potential impact of confounding factors, we meticulously considered both the patients' underlying complications and other distinct characteristics. With regard to complications (osteoporosis, hypertension, diabetes and average number of other complications), our analysis revealed no significant differences between the DJF and non‐DJF groups. Likewise, when assessing other patient characteristics such as gender and BMI, no substantial disparities were observed between the two groups. It is also noteworthy that neither of the patients who encountered perioperative complications progressed to develop distal junctional failure (DJK). This observation serves to imply that the occurrence of perioperative complications does not seem to be closely associated with the subsequent emergence of DJK.

### Selection of LIV

The ideal surgical strategy for DTH remains controversial, including selection of fusion level, especially in the selection of LIV, and previous publications have reported that selection of LIV was a risk factor for DJF.^7^, ^27^ Ailon *et al*. advocated that sacropelvic fixation should be considered for any fusion that extends proximally to T12 or above,^4^ Cecchinato *et al*. utilized short‐segment fusion with anterior corpectomy to treat post‐traumatic thoracolumbar deformity.^6^ Tezeren and Kuru also advocated^28^ the stabilization of two vertebrae above and below the fracture vertebrae could reduce implant failure. Since Cho *et al*.^11^ reported that LIV stopped at the SSV could prevent DJK, many authors have reported similar results.^5^, ^12^, ^13^ Wang *et al*.^5^ reported that fusion to the SSV could limit the development of distal junctional mechanical complications in thoracolumbar kyphosis secondary to late OVCF.

In the present study, 27, 25, and 26 patients were assigned to the SSV−, SSV, and SSV+ groups respectively, and pattern of osteotomy (PSO, modified grade 4 osteotomy and PCO) among these groups revealed no significant differences. Although there are significant differences of BMD *T*‐value, fusion levels, operation time, estimated blood loss and radiographic parameters among these groups, the incidence of DJF showed no statistically significant differences (Table 4), which indicated that fusion to SSV or more caudally could not inhibit the occurrence of DJF. Meanwhile, we found that the SSV− group had shorter fusion levels, shorter operation time, less blood loss, larger preoperative GK, while the correction rate of GK and incidence of DJF did not show significant differences compared with other groups (Table 4). This suggested that the SSV− group had the same outcome compared with other groups, but with less surgical trauma and shorter surgical duration. 3 cases with DJF required revision surgery, which could be assigned to 1 case each to group SSV−, group SSV, and group SSV+. The reoperation rate among these 3 groups was nearly the same, which indicated that the selection of fusion LIV makes little difference to the occurrence of revision surgery. Post‐SSV moved cranially compared to pre‐SSV, and LIV‐PSVL increased significantly, indicating that post‐SSV was not consistent with pre‐SSV after corrective surgery. Thus, choosing an LIV based on preoperative parameters may be inappropriate. 16 of the 25 cases in the SSV− group had LIV chosen at Apex + 2, and the number of vertebrae that LIV were away from the Apex revealed no statistical differences between the DJF and non‐DJF groups (Table 3).

We recommend fixation of the two vertebrae below the apex vertebrae for DTH (Figure 3). This strategic approach not only serves to minimize surgical trauma but also aims to yield similar outcomes compared with other strategies. It is noteworthy that, currently, a comparative assessment of the outcomes between this proposed fixation strategy and established benchmarks is lacking. However, this gap is a key focus of our upcoming research. Our future investigations aim to shed light on the potential benefits of this fixation strategy.

Berjano *et al*. determined that clinical studies looking for risk factors and solving the DJK seemed unlikely, like what we learned from PJK.^8^ In our study, 9 of 13 cases (69.2%) had screw pull‐out or loosening at the LIV, which indicated that osteoporosis plays an important role in the development of DJF (Figure 2); thus, enhancement of screws in the LIV may be beneficial for preventing DJF, theoretically. Wang *et al*.^5^ utilized cement‐augmented pedicle screws to increase pull‐out strength in osteoporotic patients, the incidence of distal junctional mechanisms in their study was 12.3%, which was a little lower than that of the present study. Further study comparing cement‐augmented pedicle screws with non‐augmented screws in the field of development of DJF was required.

### Limitations

The present study does have certain limitations that warrant consideration. First, this investigation is retrospective in nature and confined to a singular clinical setting. The retrospective design inherently harbors certain drawbacks, potentially giving rise to incomplete data, information gaps, and plausible concerns about data integrity. In order to attain a more robust foundation of evidence, the future inclusion of multicenter prospective studies is imperative. Second, the cohort is characterized by heterogeneity, which could have exerted an impact on the findings. The enrolled patients span a spectrum of etiologies, encompassing a broad age range, different follow‐up durations, and a variety of surgical interventions. As a result, a certain level of heterogeneity exists in our study, potentially introducing bias that could influence the outcomes. Lastly, the global alignment and proportion (GAP) score^29^ was not employed to predict mechanical complications because its reliability remains controversial.^30^ It is essential to acknowledge these limitations in the interpretation of the study's findings, while also recognizing that they underscore the potential directions for further research that could enhance our understanding of the intricacies surrounding DJF and its multifactorial associations.

## Conclusion

DTH may develop due to underlying etiologies such as OVCF, SK and DDD. Within the purview of our investigation, we ascertained that the incidence of DJF among DTH patients amounted to 16.7%. Age, CT HU, follow‐up, estimated blood loss, and preoperative SVA were independent risk factors for the occurrence of DJF. Prolonged follow‐up exhibited an incremental association with DJF incidence, prompting requisite long‐term monitoring.

In light of our findings, we recommend fixation of two vertebrae below the apex vertebrae for DTH. This approach, while attenuating surgical trauma, concurrently ensures similar in clinical outcomes compared with other surgical approaches. Consequently, its implementation has the potential to mitigate the incidence of DJF and heighten the overall success of surgical interventions. Our findings may provide reference for the development of clinical protocols and guidelines. Further research and clinical validation are warranted to consolidate our recommendations and facilitate their integration into routine practice, ultimately benefiting individuals with DTH.

## Conflict of Interest Statement

The authors declare that they have no affiliations with or involvement in any organization or entity with any competing interest inthe subject matter or materials discussed in this manuscript.

## Author Contributions

All the authors have made appropriate contributions to this article. Yongqiang Wang: data measurements, study design, statistical analysis and manuscript revision; Junyu Li: data measurements, study design, statistical analysis and manuscript revision; Yu Xi: statistical analysis and manuscript revision; Others: surgery and study design.

## Ethics Stement

All methods were performed in accordance with the relevant guidelines and regulation. Approval was grantedby the Peking University Third Hospital Medical Science Research Ethics Committee prior to the study. Formal consent was not required due to the retrospective design.
